# Inflammatory Pericardial Pseudocyst Secondary to Atrial Myocardial Perforation: A Rare Complication following Transvenous Pacemaker Implantation

**DOI:** 10.1155/2018/2527413

**Published:** 2018-07-03

**Authors:** Mohammad Mowaswes, Tawfik Khoury, Ziv Lahav, Ashraf Sanduka, Ayelet Shapira-Daniels, Oz M. Shapira

**Affiliations:** ^1^Department of Internal medicine, Hadassah Hebrew University Medical Center, Jerusalem, Israel; ^2^Department of Pathology, Hadassah Hebrew University Medical Center, Jerusalem, Israel; ^3^Department of Cardiothoracic Surgery, Hadassah Hebrew University Medical Center, Jerusalem, Israel

## Abstract

Pericardial cyst is an uncommon clinical-pathological entity, most often a congenital condition. We describe a case of an acquired iatrogenic pericardial pseudocyst following permanent pacemaker implantation secondary to atrial myocardial perforation. Diagnosis was achieved by a plain chest film, echocardiography, and computed tomography and confirmed intraoperatively. The pseudocyst was resected via a midline sternotomy approach. The patient recovered uneventfully. In a follow-up of 18 months, the patient is doing well.

## 1. Introduction

Pericardial cyst is an uncommon clinical-pathological entity with an estimated occurrence rate of 1 in 100,000 inhabitants [1]. Mostly, the etiology is congenital. Acquired causes include inflammatory disorders, rheumatic pericarditis, and infections such as tuberculosis and following cardiac surgery [2]. Most pericardial cysts are asymptomatic. Clinical symptoms are related to complications such as bleeding, infection, and rupture. Very rare complications include congestive heart failure, arrhythmias such as atrial fibrillation, cardiac tamponade, ventricular outflow obstruction, and sudden cardiac death [1]. Permanent transvenous pacemaker insertion is a safe procedure with a low complication rate [3]. We herein describe for an iatrogenic inflammatory pericardial pseudocyst after a permanent pacemaker insertion.

## 2. Case Report

**Figure 1:**
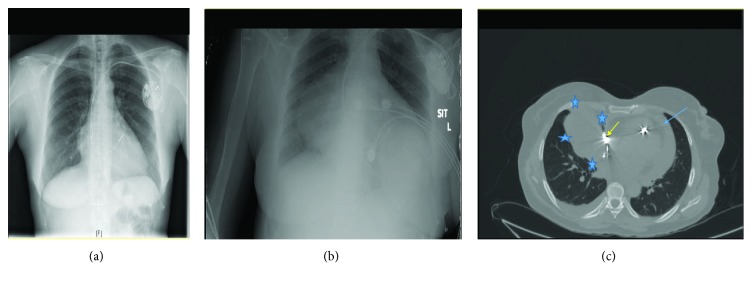
(a) Chest X-ray immediately following pacemaker insertion procedure showing normal right atrial contour with no opacification in the right lung field. (b) Chest X-ray showing opacity in the right middle lobe on admission. (c) Computed tomography showed a thickened pericardium and pericardial effusion (blue arrow), pericardial cyst (blue stars), and the pacemaker electrode lining the epicardial surface of the right ventricle (yellow arrow).

**Figure 2:**
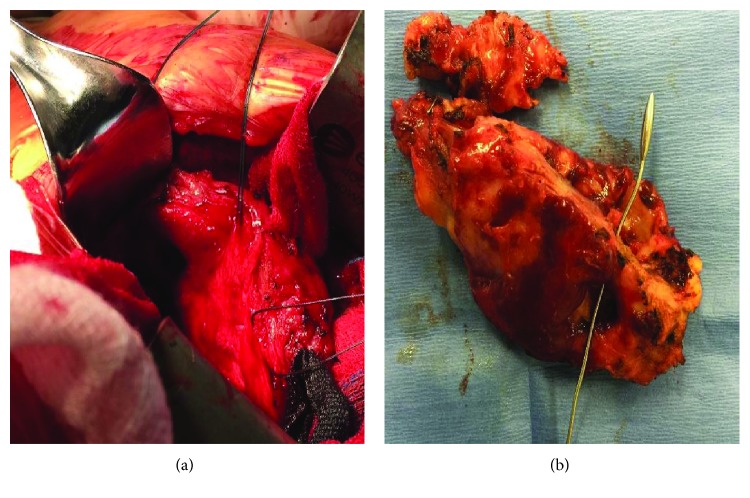
Intraoperative images during resection of the pericardial surgery and the resected pericardial cyst.

**Figure 3:**
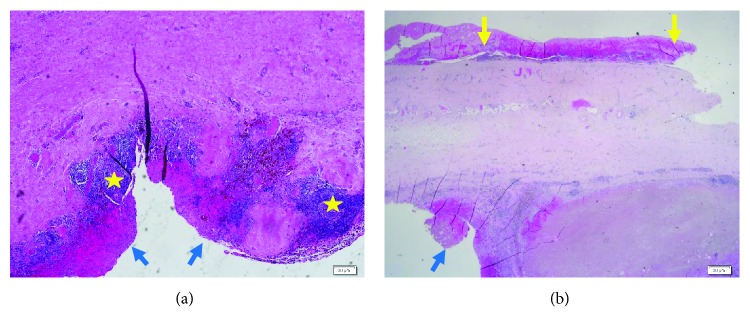
Microscopic description of the inflamed pericardial pseudocyst. (a) The inner cyst wall was devoid of the epithelial lining with ulceration and hemorrhage (blue arrows) on a background of chronic inflammation (yellow stars). (b) The outer cyst surface showed fibrinous exudate (yellow arrows).

A fifty-year-old female patient presented with a one month history of dry cough and dyspnea. One year prior to this admission, she underwent a permanent pacemaker implantation for idiopathic third-degree atrioventricular block. Twenty days after pacemaker insertion, she returned to the hospital with a burning chest pain. Transthoracic echocardiography (TTE) demonstrated a small amount of pericardial effusion. A diagnosis of pericarditis secondary to pacemaker insertion was made, and she was treated with colchicine. Repeat echocardiogram showed persistent small pericardial effusion. The patient was asymptomatic until one month prior to this admission when she developed dyspnea, dry cough, and fever. Vital signs on admission were respiratory rate of 20/minute, blood pressure 109/66 mmHg, heart rate 121/minute, and temperature 37.1°C. Room air oxygen saturation was 95%. Otherwise, her physical examination was unremarkable. Heart sounds were normal with no murmurs, rub, or other abnormal sounds. Chest film showed a round opacity with smooth borders in the midright lung field—a major change compared to prior films (Figures 1(a) and 1(b)). Blood tests were remarkable for white blood cell count of 15,000 · 10*E*9/L and elevated C-reactive protein (CRP) of 6.25 mg/dL. Computed tomography (CT) of the chest revealed thickened pericardium with pericardial effusion (Figure 1(c), blue arrow), a 6.8 × 6.2 cm thick-walled pericardial cystic mass (blue stars) adjacent to the tip of atrial pacemaker lead suspected to be extracardiac (yellow arrow). TTE showed moderate amount of pericardial effusion and the cystic mass. Given her clinical presentation and the differential diagnosis, we elected to excise the mass. The chest was opened via a midsternotomy incision. We found a large (7.0 × 2.5 × 0.7 cm) cystic mass. Macroscopically, the cyst wall was thickened with intense fibrosis, areas of hemorrhages, and active inflammation (Figure 2). The orifice of the cyst was opposite the tip of the atrial pacemaker lead, which was covered by intense fibrosis. The cystic mass was completely excised, carefully preserving the right phrenic nerve. Histological examination revealed a lack of the epithelial lining over the inner surface, scattered ulcerations, fibrin depositions, hemorrhages, hemosiderin-laden macrophages, and chronic inflammation (Figure 3). The patient recovered uneventfully. In a follow-up of 18 months, the patient is asymptomatic in functional class I. Follow-up plain chest film is normal. Repeat echocardiogram showed no pericardial pathology.

## 3. Discussion

Primary pericardial cyst is an uncommon congenital clinical-pathological entity with a benign clinical course. Rare complications of benign pericardial cysts include rupture, bleeding, and compression of adjacent structures. Surgical removal of the cyst is indicated for very large-sized cysts, when complication occurs or when the diagnosis is uncertain [2].

Pacemaker insertion is a safe procedure with a 3% incidence of major complications, including perforation of heart chambers with or without tamponade, pneumothorax, hemothorax, and air embolism [4]. In the present case, diagnosis of early postpacemaker implantation pericarditis was established by clinical presentation, laboratory data, and echocardiography. A normal plain film of the chest immediately after pacemaker implantation, the close proximity of the pacemaker lead to the orifice of the pseudocyst, and the histopathological findings are all highly suggestive that repetitive mechanical trauma of the pericardium by the perforated atrial pacemaker lead caused fibrinous pericarditis followed by an inflammatory pericardial pseudocyst. Being unaware of this unique clinical-pathological entity, we elected to explore the chest, excise the lesion to establish a diagnosis in a young patient with a rapidly enlarging mediastinal mass, and manage the perforated atrial lead. Complete excision of the pseudocyst was achieved. The perforated pacemaker electrode tip was sealed by an intense fibrotic response and therefore was left in place.

In conclusion, inflammatory pericardial pseudocyst, most likely, due to repetitive trauma by a perforated lead, is a rare complication of transvenous pacemaker implantation. It should be included in the differential diagnosis of postprocedural enlarging cystic mediastinal mass.
